# NR2F6 Expression Correlates with Pelvic Lymph Node Metastasis and Poor Prognosis in Early-Stage Cervical Cancer

**DOI:** 10.3390/ijms17101694

**Published:** 2016-10-20

**Authors:** Chunhao Niu, Xiaoying Sun, Weijing Zhang, Han Li, Liqun Xu, Jun Li, Benke Xu, Yanna Zhang

**Affiliations:** 1 State Key Laboratory of Oncology in South China, Collaborative Innovation Center for Cancer Medicine, Department of Gynecologic Oncology, Cancer Center, Sun Yat-sen University, No. 651, Dongfeng Road East, Guangzhou 510060, China; niuchh@sysucc.org.cn (C.N.); sunxiaoy@sysucc.org.cn (X.S.); zhangwj@sysucc.org.cn (W.Z.); lihan@sysucc.org.cn (H.L.); 2 Department of Gynecology, Women and Children Hospital of Guangdong Province, No. 13, Guang Yuan Road, Guangzhou 510060, China; xuliqunyang@126.com; 3 Department of Biochemistry, Zhongshan School of Medicine, Sun Yat-sen University, Guangzhou 510080, China; lijun37@mail.sysu.edu.cn; 4 Department of Anatomy, Medical School of Yangtzeu University, Jingzhou 434000, China

**Keywords:** NR2F6 (nuclear receptor subfamily 2 group F member 6), cervical cancer, lymph node metastasis, prognosis, biomarker

## Abstract

Background: There is an abnormal expression of nuclear receptor subfamily 2 group F member 6 (NR2F6) in human cancers such as breast cancer, colon cancer, and acute myelogenous leukemia. However, its clinical significance in cervical cancer has not been established. We explored NR2F6 expression and its clinicopathological significance in early-stage cervical cancer. Methods: NR2F6 expression in cervical cancer cell lines and cervical cancer tissues was determined by Western blotting, real-time PCR, and immunochemistry (IHC). NR2F6 expression in 189 human early-stage cervical cancer tissue samples was evaluated using IHC. The relevance between NR2F6 expression and early-stage cervical cancer prognosis and clinicopathological features was determined. Results: There was marked NR2F6 mRNA and protein overexpression in the cervical cancer cells and clinical tissues compared with an immortalized squamous cell line and adjacent noncancerous cervical tissues, respectively. In the 189 cervical cancer samples, NR2F6 expression was positively related to International Federation of Gynecology and Obstetrics (FIGO) stage (*p* = 0.006), squamous cell carcinoma antigen (*p* = 0.006), vital status (*p* < 0.001), tumor recurrence (*p =* 0.001), chemotherapy (*p =* 0.039), and lymph node metastasis (*p* < 0.001). Overall and disease-free survival was shorter in patients with early-stage cervical cancer and higher NR2F6 levels than in patients with lower levels of NR2F6. Univariate and multivariate analysis determined that NR2F6 was an independent prognostic factor of survival in early-stage cervical cancer. Conclusions: Taken together, our findings suggest that high NR2F6 expression predicts pelvic lymph node metastasis, tumor recurrence and poor prognosis in early-stage cervical cancer. NR2F6 might be a novel prognostic biomarker and potential therapeutic target of cervical cancer.

## 1. Introduction

Cervical cancer is the fourth most commonly diagnosed malignancy and the fourth leading cause of cancer-related death in females worldwide, with an estimated global incidence of 527,600 new cases and 265,700 deaths annually [1]. It is caused by high-risk human papillomavirus (HPV), particularly type 16 (HPV16) and 18 (HPV18), which account for approximately two-thirds of all cervical cancer cases [2]. Unlike most other gynecological malignancies, cervical cancer is clinically staged preoperatively [3]. Radical hysterectomy, plus lymphadenectomy (RH) or chemoradiation, are recommended for early-stage cervical cancer (International Federation of Gynecology and Obstetrics (FIGO) stage IB–IIA) [4]. Moreover, postoperative patients with high or intermediate recurrence risk factors require further adjuvant treatments [5]. Lymph node metastasis (LNM) is an important high-risk factor of recurrence and a strong predictor of poor prognosis in early-stage cervical cancer [5,6]. The mortality of patients with LNM is higher than that in patients without LNM [7]. Therefore, identifying novel and special markers for LNM early detection and prediction in cervical cancer would be of great clinical value.

Nuclear receptor subfamily 2 group F member 6 (NR2F6), also designated EAR-2, EAR2 or ERBAL2, is located on chromosome 19p13.1.1; it encodes a highly conserved 43-kDa protein. NR2F6 belongs to the chicken ovalbumin upstream promoter-transcription factors (COUP-TFs), an NR2 member [8]. COUP-TFs comprise three members: COUP-TFI (EAR3, NR2F1), COUP-TFII (ARP-1, NR2F2), and NR2F6, and regulate many key biological processes such as organogenesis, neurogenesis, and cellular differentiation during embryonic development [9,10,11,12]. NR2F6 is a negative regulator of the human luteinizing hormone receptor and renin gene transcription [13,14] and is a negative modulator of T cell development [15]. Recently, NR2F6 overexpression was reported in several human tumors, and it may be related to differentiation, apoptosis, and survivability [16,17]. NR2F6 is more highly expressed in breast cancer than in normal breast tissue; NR profiling with the NCI-60 cancer cell panel indicates that cells with lower NR2F6 expression exhibit higher sensitivity to anti-cancer drugs targeting microtubules [12,18]. NR2F6 knockdown by RNA interference (RNAi) induces colon cancer cell apoptosis by inhibiting the X-linked inhibitor of apoptosis protein (XIAP) expression [17]. In addition, NR2F6 expression is >4-fold in acute myeloid leukemia (AML) cells with growth ability than in cells without it; NR2F6 knockdown induces AML cell terminal differentiation and apoptosis [16]. These results suggest that NR2F6 may play different roles in various cancers. However, NR2F6 expression characteristics and its clinical significance in cervical cancer remain unknown.

Herein, we explored the NR2F6 expression characteristics in cervical cancer cell lines and early-stage cervical cancer tissues. We also investigated the relationship between NR2F6 protein expression and the clinical manifestations and survival outcome of 189 early-stage cervical cancer cases.

## 2. Results

### 2.1. Nuclear Receptor Subfamily 2 Group F Member 6 (NR2F6) Is Upregulated in Cervical Cancer Cell Lines

**Figure 1:**
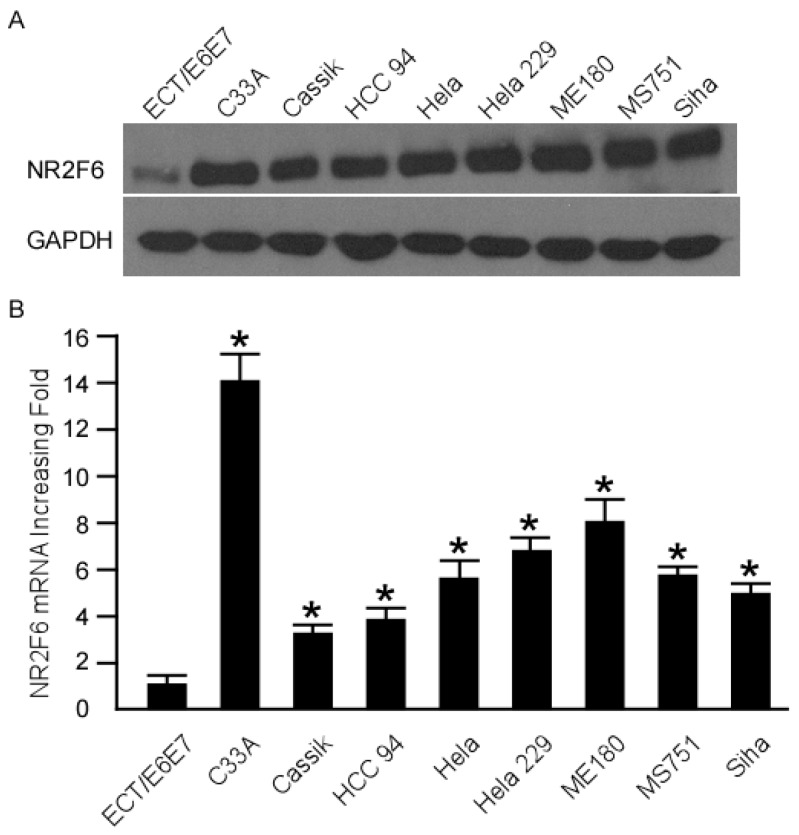
NR2F6 (nuclear receptor subfamily 2 group F member 6) mRNA and protein overexpression in cervical cancer cell lines (C33A, CaSSIK, HCC 94, Hela, Hela 229, ME 180, MS 751, Siha) compared with human cervical immortalized squamous cell line Ect1/E6E7. Western blot (**A**) and real-time PCR (**B**) investigation of NR2F6 mRNA and protein expression levels in cervical cancer cell lines and the Ect1/E6E7 cell line. Expression levels were normalized against GAPDH (the housekeeping gene glyceraldehyde-3-phosphate dehydrogenase, internal control). Error bars: standard deviation (SD) of the mean from three parallel experiments. * *p* < 0.05.

NR2F6 mRNA and protein expression were overexpressed in all cervical cancer cell lines as compared to the Ect1/E6E7 cell line (Figure 1A,B), indicating that NR2F6 is upregulated in cervical cancer cell lines.

### 2.2. NR2F6 Is Upregulated in Cervical Cancer Tissues

**Figure 2:**
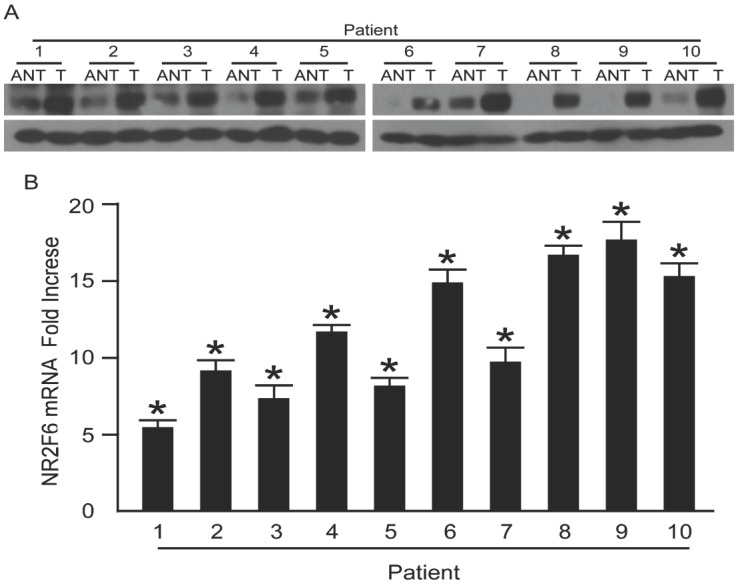

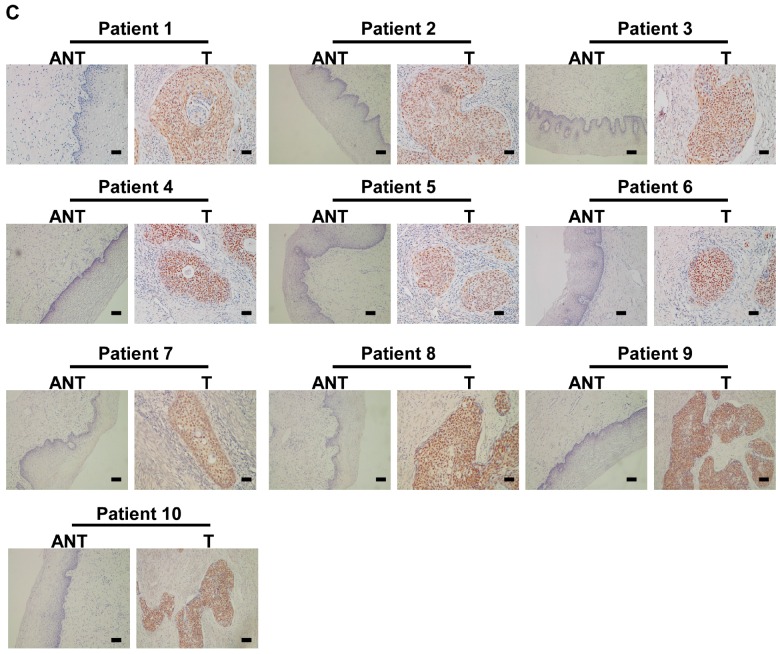
NR2F6 mRNA and protein overexpression in cervical cancer tissues. (**A**) Representative Western blots of NR2F6 protein expression in 10 matched pairs of cervical cancer tissues (T) and adjacent noncancerous tissues (ANT). GAPDH was used as the loading control; (**B**) Real-time PCR quantification of average T/ANT ratios of NR2F6 mRNA expression in paired cervical cancer (T) and adjacent noncancerous tissues (ANT) and normalization against GAPDH. Error bars, standard deviation (SD) of the mean calculated from three parallel experiments; (**C**) Immunohistochemical (IHC) assay of NR2F6 protein expression in 10 pairs of matched cervical cancer tissues. Scale bars = 50 μm, * *p* < 0.05.

Western blot and real-time PCR were performed on 10 paired cervical carcinoma tissues and the adjacent noncancerous tissues to determine NR2F6 expression in the cervical cancer tissues. NR2F6 mRNA and protein expression were upregulated in all cervical cancer samples compared to the matched adjacent noncancerous samples (Figure 2A,B). The IHC analysis results were consistent with the above findings (Figure 2C), indicating that NR2F6 is upregulated in cervical cancer tissues.

### 2.3. Correlation between NR2F6 Overexpression and Cervical Cancer Clinical Features

**Figure 3:**
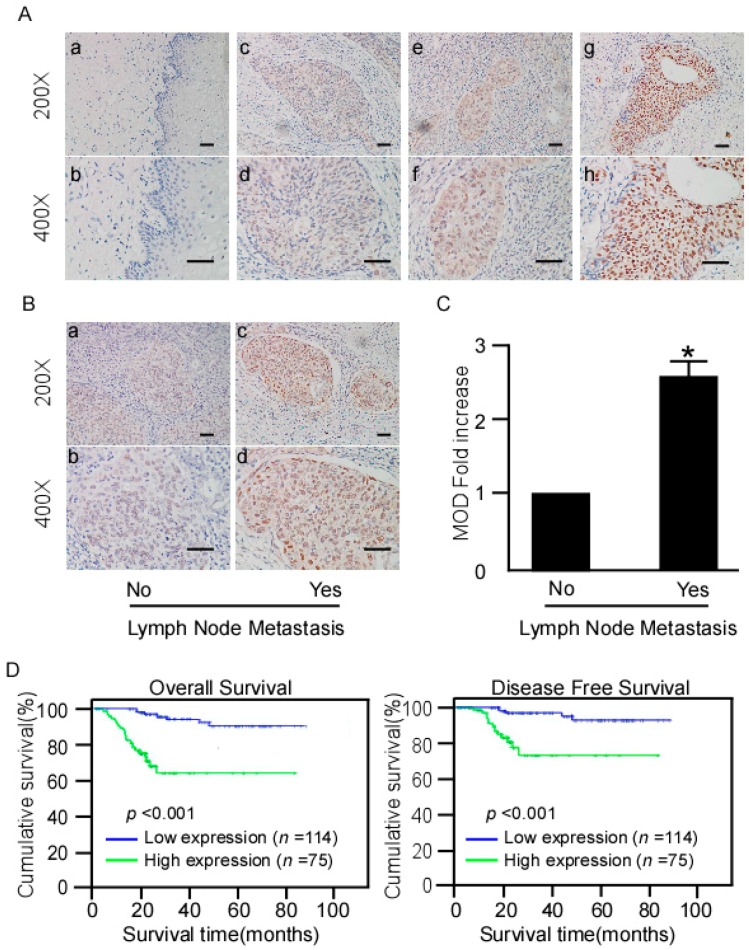
IHC detection of NR2F6 protein expression in paraffin-embedded cervical cancer tissues. Positive NR2F6 staining was observed mainly in the nuclei. (**A**) (**a**,**b**) NR2F6 expression was not detected in normal cervical tissues; (**c**,**d**) representative images of weak NR2F6 staining in cervical cancer tissues; (**e**,**f**) representative images of moderate NR2F6 staining in cervical cancer tissues; (**g**,**h**) representative images of strong NR2F6 staining in cervical cancer tissues(scale bars = 50 μm); (**B**,**C**) Statistical analyses of the average MOD (mean optical density) of NR2F6 staining in the LNM (Lymph node metastasis) and LNM-free groups. Scale bars = 50 μm, * *p* < 0.05; (**D**) Kaplan–Meier curves of univariate analysis data (log-rank test) showing the OS (overall survival) and DFS (disease-free survival) for patients with high versus low NR2F6 expression.

To confirm whether NR2F6 protein overexpression is correlated with cervical cancer clinicopathological features, IHC analysis was performed on 189 cervical cancer tissue samples that included FIGO stage IB1 (72 cases, 38.1%), stage IB2 (37 cases, 19.6%), stage IIA1 (54 cases, 28.5%), and stage IIA2 (26 cases, 13.8%) disease. Seventy-five samples (39.7%) had high NR2F6 protein expression; staining was weakly positive or negative in 114 samples (60.3%, Table 1). We observed specific NR2F6 staining mainly in the cancer cell nuclei (Figure 3A). Positive NR2F6 protein expression was noted in 27.8% (20/72), 37.8% (14/37), 53.7% (29/54), and 46.2% (12/26) of tumors at FIGO stage IB1, IB2, IIA1, and IIA2, respectively (*p* < 0.05, χ^2^ test) (Table 2). Quantitative IHC analysis showed that the mean optical density (MOD) of NR2F6 staining in the LNM group was higher than that in the LNM-free group (*p* < 0.01, Figure 3B,C).

NR2F6 protein expression was markedly correlated with FIGO stage, squamous cell carcinoma (SCC) antigen, vital status, chemotherapy, tumor recurrence, and most importantly, LNM (Table 2). Spearman’s correlation analysis confirmed that high NR2F6 expression correlated with these data (Table 3). However, there were no significant correlations between NR2F6 protein expression and age, HPV infection, histological differentiation grade, histological type, myometrium invasion, surgical margin properties, lymphovascular space involvement, parauterine organ infiltration, or radiation.

### 2.4. High NR2F6 Expression Is Correlated with Poor Prognosis in Early-Stage Cervical Cancer

**Figure 4:**
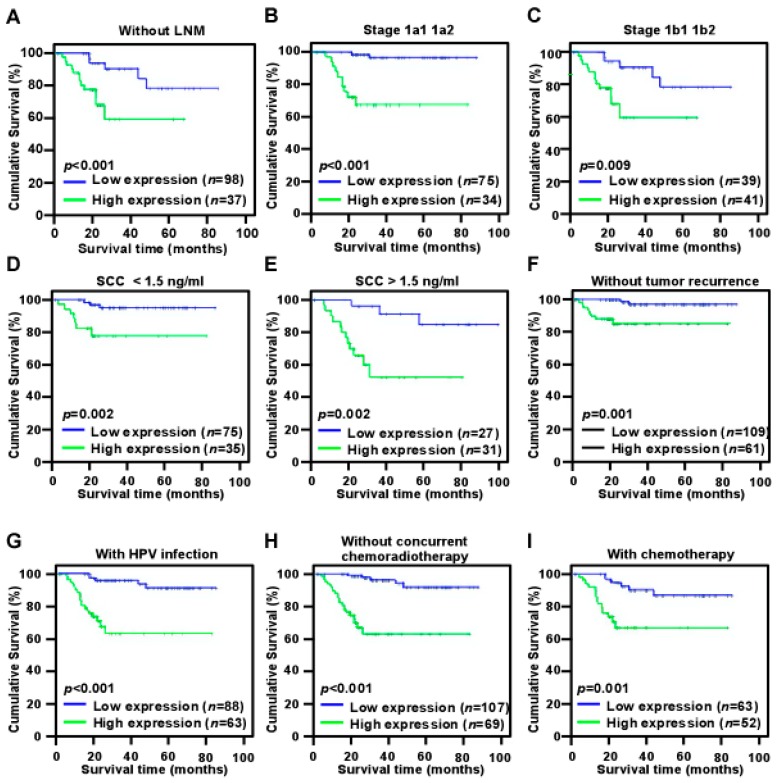
Kaplan–Meier curves of univariate analysis data (log-rank test) of patients with high versus low NR2F6 expression. (**A**) OS of LNM-free patients; (**B**) OS of patients with stage IIA1–IIA2 disease; (**C**) OS of patients with stage IB1–IB2 disease with high versus low NR2F6 expression; (**D**) OS of patients with SCC (squamous cell carcinoma antigen) antigen <1.5 ng/mL; (**E**) OS of patients with SCC antigen >1.5 ng/mL; (**F**) OS of patients without recurrence; (**G**) OS of patients with HPV infection; (**H**) OS of patients without CCRT (concurrent chemoradiotherapy); (**I**) OS of patients who received chemotherapy.

Log-rank testing showed that patients with high NR2F6 expression had significantly shorter survival (Figure 3D), whereas patients with low NR2F6 expression had significantly longer survival (Figure 4, log-rank, *p* < 0.001). The cumulative rates of OS and disease-free survival (DFS) were 93.9% and 95.6%, respectively, in the low-NR2F6 group, and were 70.7% and 81.3%, respectively, in the high-NR2F6 group. We also evaluated the prognostic value of NR2F6 expression in patient subgroups stratified by age, SCC antigen, HPV infection, tumor size, differentiation grade, FIGO stage, pelvic LNM (PLNM), radiation, chemotherapy, concurrent chemotherapy and radiotherapy, surgical margin properties, lymphovascular space involvement, myometrium invasion, and parauterine organ infiltration. In patients without PLNM, patients with high NR2F6 expression had significantly shorter OS than patients with low NR2F6 expression (log-rank, *p* < 0.001) and those with FIGO stage IB1–IB2 disease (log-rank, *p* < 0.001), FIGO stage IIA1–IIA2 disease (log-rank test, *p* < 0.01), SCC antigen ≤1.5 ng/mL (log-rank, *p* < 0.01), SCC antigen ≥1.5 ng/mL (log-rank test, *p* < 0.01), without tumor recurrence (log-rank test, *p =* 0.01), HPV infection (log-rank test, *p* < 0.01), not receiving concurrent chemotherapy and radiotherapy (log-rank test, *p* < 0.001), and receiving chemotherapy (log-rank, *p* = 0.001) (Figure 4). Univariate Cox regression analyses revealed that NR2F6 protein level, SCC antigen, PLNM, and recurrence were significant prognostic factors (Table 4). Multivariate Cox regression analysis determined that NR2F6 protein level and recurrence were independent prognostic markers for early-stage cervical cancer (Table 4). Our findings showed that NR2F6 may be a promising prognostic indicator for early-stage cervical cancer.

## 3. Discussion

To our knowledge, this is the first report that high NR2F6 protein expression is associated with poor prognosis and clinical characteristics, especially PLNM, in early-stage cervical cancer. Moreover, OS and DFS were decreased in patients with high NR2F6 expression, particularly those who received chemotherapy. Multivariate analysis suggested that NR2F6 expression might be an independent prognostic marker of survival in early-stage cervical cancer. Taken together, our findings suggest that NR2F6 may be a promising novel biomarker and therapeutic target for treating early-stage cervical cancer.

Recent studies have indicated that NR2F6 is overexpressed in patients with certain cancers. NR2F6 overexpression is related to tumor aggressiveness in leukemia and colon cancer [16,17]. These reports indicate that NR2F6 may be a vital element that promotes and accelerates cancer development. However, its role in cervical cancer is unknown. Therefore, we assessed whether NR2F6 upregulation is clinically associated with cervical cancer development and progression. In the present study, NR2F6 mRNA and protein were upregulated in cervical cancer cell lines and cervical cancer tissue (stage IB–IIA). Furthermore, IHC showed a significant positive correlation between NR2F6 expression and FIGO stage, SCC antigen, tumor recurrence, vital status, chemotherapy, and LNM. These results indicate that NR2F6 plays an important role in cervical cancer progression and may be an identification biomarker for patients with a more aggressive form of cervical cancer. Univariate and multivariate analyses revealed that NR2F6 expression might be an independent prognostic predictor of poor prognosis in early-stage cervical cancer.

LNM is an important prognostic factor and the key to determining treatment for early-stage cervical cancer [7,19,20]. So far, standard treatment for early-stage cervical cancer remains RH or chemoradiation [21]. The recommended treatment for early-stage cervical cancer without clinical LNM is RH. If the postoperative lymph nodes are tumor-positive, patients are offered chemoradiation, which would render the initial surgery redundant in hindsight. Furthermore, surgery plus chemoradiation is associated with severe morbidity. Predicting the presence of LNM before treatment would enable the consideration for primary chemoradiation, which is equally effective but is associated with a different treatment-related morbidity pattern [7]. Consequently, it is important to predict LNM before treatment. However, a specific preoperative marker for predicting LNM that would prevent unnecessary surgery and decrease severe morbidity has not been established. In the present study, we failed to show that PLNM is an independent prognostic factor of OS. As all patients involved had early-stage disease, few had PLNM and their prognosis was good. However, univariate Cox regression analyses determined that the significant prognostic factors included PLNM. We also found that NR2F6 was strongly positively associated with PLNM. Lastly, we found that high NR2F6 protein expression was significantly correlated with shorter OS in the LNM-free subgroup, which suggests that NR2F6 might be a potential predictive biomarker of poor OS in patients with cervical cancer without LNM. However, further studies are needed to verify our results in a larger cervical cancer cohort with LNM and to investigate the mechanism of NR2F6 promotion of LNM in cervical cancer.

Since the Pap smear screening program—an effective early cervical cancer detection method—became widespread, early-stage diagnosis is increasing; however, the recurrence risk after treatment in early-stage cervical cancer remains at 15%–30% [20,22,23,24]. Cervical cancer recurrence is accompanied by poor prognosis. Furthermore, there are few feasible treatment options [25]. To reduce the risk of recurrence, adjuvant treatment that may produce severe acute complications is recommended [26,27,28]. Consequently, a marker that predicts recurrence is important for counseling patients and determining adjuvant treatment. In our cohort, there was a strong correlation between NR2F6 protein expression and tumor recurrence. Univariate and multivariate Cox regression analyses revealed that tumor recurrence was an independent prognostic marker of OS in cervical cancer. A more detailed survival study showed that shorter OS and high NR2F6 expression were significantly correlated in the no tumor recurrence subgroup. These results suggest that NR2F6 might be a potential predictive marker of poor OS of cervical cancer without tumor recurrence.

Further treatments are required for postoperative patients with high or intermediate risk factors for recurrence. Adjuvant therapies after RH remain disputed. Various guidelines, including those of the National Cancer Institute [29], National Comprehensive Cancer Network [30], and the European Society of Medical Oncology [31], recommend concurrent chemoradiotherapy (CCRT) or radiation therapy (RT) as the standard adjuvant treatment after RH. However, several recent retrospective studies have indicated that, for patients with intermediate risk factors, adjuvant chemotherapy (cisplatin (CDDP) based combination regimens) alone had at least equal survival effects compared with postoperative RT [32,33,34,35,36,37]. In our study, patients with high or intermediate risk factors, i.e., PLNM, positive parametrial involvement, positive surgical margin, deep stromal invasion, positive lymphovascular space involvement, large tumor (>4 cm), and high differentiation grade, received chemotherapy and/or radiotherapy. Furthermore, patients with only a positive surgical margin or deep stromal invasion received radiotherapy. Patients with only a large tumor (>4 cm), lymphovascular space involvement, or high a differentiation grade received chemotherapy. Interestingly, NR2F6 protein expression was correlated with chemotherapy. However, no correlation was observed in the concurrent chemotherapy and radiotherapy or radiotherapy-only subgroups. Furthermore, our results showed that higher NR2F6 expression and significantly shorter OS were correlated in the chemotherapy subgroup but were not associated in the postoperative radiotherapy or postoperative concurrent chemotherapy and radiotherapy subgroups. Therefore, our results demonstrate that NR2F6 expression may be a more significant predictor of the prognosis for patients with early-stage cervical cancer who require chemotherapy.

Recently, using the immune system to elicit anti-tumor immune responses has been considered a promising cancer treatment. T cells are one of the most important immune cells for inhibiting tumors [38]. Blocking the immune system inhibitory pathways, such as the cytotoxic T lymphocyte-associated protein 4 (CTLA-4) and programmed cell death 1 (PD-1)/PD-1 ligand (PD-L1) pathways, considered promising novel therapeutic advances, have been successfully introduced to the clinic. CTLA-4 and PD-1/PD-L1 are also termed immune checkpoints. Hermann-Kleiter et al. demonstrated that NR2F6 acts as an intracellular immune checkpoint in T cells. Furthermore, they found that NR2F6 deletion has the same effect as blocking PD-1/PD-L1 interaction, considered an established immune checkpoint mechanism. They also found that NR2F6 directly represses the transcription of cytokine genes in T cells related to cancer cell rejection, such as interleukin (IL)-2, tumor necrosis factor (TNF)-α, and interferon (IFN)-γ [39]. Therefore, NR2F6 may be a potential cancer therapeutic target. We demonstrate that NR2F6 is upregulated in cervical cancer, and is correlated with PLNM, tumor recurrence, and poor prognosis in early-stage cervical cancer. Therefore, we hypothesize that NR2F6 might be a therapeutic target in early-stage cervical cancer.

## 4. Materials and Methods

### 4.1. Patient Information and Tissue Specimens

The present study was conducted using 189 paraffin-embedded early-stage cervical cancer specimens archived at the Sun Yat-sen University Cancer Center Department of Pathology (Guangzhou, China) from 2007 to 2009. Ten paired early-stage cervical cancer and adjacent normal tissues were collected from patients who had undergone surgery from January 2015 to May 2016 at our center. The selection criteria were: (1) Had undergone RH without preoperative chemotherapy, radiotherapy, or hormonal therapy; (2) Confirmed as FIGO stage IB1–IIA2 according to the 2009 FIGO criteria; (3) Only had cervical cancer; (4) Clinical data were complete; (5) Had over 5 years’ follow-up records and the deadline as January 2014. The patient clinical information is summarized in Table 1. The follow-up time for the primary cervical cancer cohort was 3–99 months; median follow-up time was 51 months.

Approval from the Sun Yat-sen University Cancer Center Institutional Review Board was obtained for the purpose of this research (YB2015-043-01, 10 December 2015). All patients provided their informed consent for the purpose of this research.

### 4.2. Cell Lines

The human cervical immortalized squamous cell line Ect1/E6E7 was obtained from Nanjing Hwatao Biopharm (Nanjing, China). Eight human cervical cancer cell lines (CaSki, C33A, HeLa, HCC 94, HeLa 299, ME-180, MS751, SiHa) were purchased from American Type Culture Collection (Manassas, VA, USA). All cell lines were cultured in an RPMI 1640 medium (Gibco BRL, Rockville, MD, USA) supplemented with 10% fetal bovine serum (HyClone Laboratories, Logan, UT, USA) and 1% antibiotics (100 μg/mL streptomycin and 100 U/mL penicillin) in a 5% humidified atmosphere with CO_2_ at 37 °C.

### 4.3. Real-Time PCR

Total cellular and fresh tissue RNA samples were extracted using TRIzol (Invitrogen, Carlsbad, CA, USA) according to the manufacturer’s instructions. For PCR amplification of complementary DNA, initial amplification using gene-specific primers was conducted with denaturation at 95 °C for 10 min, followed by 30 denaturation cycles at 95 °C for 60 s, primer annealing at 55 °C for 30 s, and primer extension at 72 °C for 30 s. During cycling completion, final extension was carried out for 5 min at 95 °C, and then the reaction mixture was held at 4 °C. The gene expression levels were normalized to that of the housekeeping gene glyceraldehyde-3-phosphate dehydrogenase (*GAPDH*, internal control). All primers were designed using Primer Express version 2.0 software (Applied Biosystems, Foster City, CA, USA). The primers used were as follows: NR2F6 forward: 5′-CGAGGGCTGCAAGAGCTTT-3′, reverse: 5′-GCACTTCTTGAGACGGCAGTACT-3′; GAPDH forward: 5′-ATTCCACCCATGGCAAATTC-3′, reverse: 5′-TGGGATTTCCATTGATGACAAG-3′.

### 4.4. Western Blotting

Protein extraction kits were used to prepare total protein according to the manufacturer’s instructions (Millipore, Billerica, MA, USA). Equal concentrations of denatured protein samples (20 μg) were separated on 9% sodium dodecyl sulfate–polyacrylamide gels. Then, the samples were transferred to polyvinylidene fluoride membranes (Immobilon-P, Millipore). After blocking with 5% non-fat milk in Tris-buffered saline/0.1% Tween 20 (TBST) for 1 h at room temperature, the membranes were incubated with specific primary antibody and washed with TBST followed by incubation with horseradish peroxidase–conjugated secondary antibody. Proteins were detected using electrochemiluminescence reagents (Amersham Pharmacia Biotech, Piscataway, NJ, USA).

### 4.5. Immunohistochemical (IHC) Analysis

IHC analysis was used to study the altered NR2F6 protein expression in the 189 paraffin-embedded samples using classic protocols. Briefly, 4-μm thick sections were baked at 60 °C for 1 h, cleared in xylene, and rehydrated. All deparaffinized sections were immersed in EDTA antigen retrieval buffer and then autoclaved at 121 °C for 4 min for antigen retrieval. The slides were incubated in 3% hydrogen peroxide for 10 min to block endogenous peroxidase activity; nonspecific binding was blocked by incubation with 1% bovine serum albumin. The sections were incubated with a mouse monoclonal antibody to NR2F6 (Upstate Biotechnology, 1:150, Proteintech, Rosemont, IL, USA, 60117-1-lg) at 4 °C overnight. Normal goat serum was used as the negative control. After cleaning with PBST, the slides were incubated with biotinylated anti-mouse secondary antibody (Sigma, St. Louis, MO, USA) at room temperature for 30 min, and then incubated with streptavidin–horseradish peroxidase complex (Sigma). The slides were immersed in 3-amino-9-ethylcarbazole, counterstained with 10% Mayer’s hematoxylin, dehydrated, and mounted in Crystal Mount.

Two pathologists reviewed and evaluated the degree of immunostaining independently, according to the proportion of positively stained tumor cells and the staining intensity. The former was scored as follows: 0 (no positive tumor cells); 1 (<10% positive); 2 (10%–50% positive); 3 (>50% positive). Staining intensity was scored as follows: 0, no staining; 1, weak staining (light yellow); 2, moderate staining (yellow brown); 3, strong staining (brown). The staining index (SI) was calculated as the staining intensity score × proportion of positive tumor cells (range 0–9). Cut-off values were selected based on the measure of heterogeneity with log-rank testing with respect to overall survival (OS). The optimal cut-off value was determined as follows: SI ≥6 was deemed high expression and SI ≤4 was deemed low expression.

### 4.6. Statistical Analysis

All statistical analyses were performed using SPSS version 19.0 (SPSS, Chicago, IL, USA). The relationship between NR2F6 expression and the clinicopathologic features of cervical cancer was analyzed using the chi-square test and Fisher’s exact test. We used Spearman’s rank correlation coefficients to calculate the bivariate correlations between the studied variables. We plotted survival curves using the Kaplan–Meier method and compared them using log-rank testing. Relative risk ratios were calculated using the Cox proportional hazard model. Univariate and multivariate survival distributions were compared using log-rank testing. We considered *p* < 0.05 statistically significant.

## 5. Conclusions

This is the first study to evaluate NR2F6 expression and its clinical significance in early-stage cervical cancer. NR2F6 might be a useful prognostic biomarker and potential therapeutic target for early-stage cervical cancer.

## Figures and Tables

**Table 1:** Clinicopathological characteristics and tumor expression of *NR2F6* in early-stage cervical cancer.

Characteristic		Number of Cases (%)
**Age (years)**	≤46	95 (50.3)
	>46	94 (49.7)
**FIGO (International Federation of Gynecology and Obstetrics) Stage**	Ib1	72 (38.1)
	Ib2	37 (19.6)
	IIa1	54 (28.6)
	IIa2	26 (13.8)
**Histological Type**	Squamous carcinoma	179 (94.7)
	Adenocarcinoma	10 (5.3)
**Tumor Size, cm**	<4 cm	154(81.5)
	≥4 cm	35 (18.5)
**Squamous Cell Carcinoma Antigen, ng/mL**	≤1.5	110 (65.5)
	>1.5	58 (34.5)
**HPV (Human Papillomavirus) Infection**	No	38 (20.1)
	Yes	151 (79.9)
**Pelvic Lymph Node Metastasis**	No	135 (71.4)
	Yes	54 (28.6)
**Tumor Recurrence**	No	170 (89.9)
	Yes	19 (10.1)
**Vital Status (at last follow-up)**	Alive	160 (84.7)
	Dead	29 (15.3)
**Differentiation Grade**	G1	73 (49.6)
	G2	41 (27.9)
	G3	33 (22.5)
**Myometrium Invasion**	<1/2	66 (34.9)
	≥1/2	122 (64.6)
**Property of Surgical Margin**	No	176 (93.1)
	Yes	13 (6.9)
**Infiltration of Parauterine Organ**	No	179 (94.7)
	Yes	10 (5.3)
**Lymphovascular Space Involvement**	No	173 (91.5)
	Yes	16 (8.0)
**Chemotherapy**	No	73 (38.6)
	Yes	115 (61.2)
**Radiation**	No	169 (89.4)
	Yes	20 (10.6)
**Concurrent chemotherapy and Radiotherapy**	No	176 (93.1)
	Yes	13 (6.9)
**Expression of NR2f6**	Low or none	114 (60.3)
	High	75 (39.7)

**Table 2:** Correlation between NR2F6 expression and the clinicopathological features of early-stage cervical cancer.

Characteristic		Total	NR2F6		Chi-Squared Test *p*-Value	Fisher’s Exact Test *p*-Value
			No or Weak Expression	Moderate or Strong Expression		
**Age (years)**	≤46	95	58 (30.7)	37 (19.6)	0.835	0.882
	>46	94	52 (29.6)	41 (20.1)		
**Histological Type**	Adenocarcinoma	10	7 (3.7)	3 (1.6)	0.520	0.742
	Squamous cell carcinoma	179	107 (56.6)	72 (38.1)		
**HPV Infection**	No	38	26 (13.8)	12 (6.3)	0.253	0.272
	Yes	151	88 (46.6)	63 (33.3)		
**FIGO Stage**	Ib1	72	52 (27.5)	20 (10.6)	0.027	-
	Ib2	37	23 (12.2)	14 (7.4)		
	IIa1	54	25 (13.2)	29 (15.3)		
	IIa2	26	14 (7.4)	12 (6.3)		
**Pelvic Lymph Node Metastasis**	Absent	135	98 (51.9)	37 (19.6)	0.000	0.000
	Present	54	16 (8.5)	38 (20.1)		
**Squamous Cell Carcinoma Antigen, ng/mL**	≤1.5	110	75 (44.6)	35 (16.1)	0.006	0.008
	>1.5	58	27(20.8)	31(18.5)		
**Tumor Size**	<4 cm	154	93 (49.2)	61 (32.3)	0.996	1.000
	≥4 cm	35	21 (11.1)	14 (17.4)		
**Tumor Recurrence**	No	170	109 (57.7)	61 (32.3)	0.001	0.002
	Yes	19	5 (2.6)	14 (7.4)		
**Vital Status (At Last Follow-up)**	Alive	160	107 (56.6)	53 (28.0)	0.000	0.000
	Dead	29	7 (3.7)	22 (11.6)		
**Differentiation Grade**	G1	73	48 (32.7)	25 (17.0)	0.113	-
	G2	41	27 (18.4)	14 (9.5)		
	G3	33	20 (13.6)	13 (8.8)		
**Chemotherapy**	No	73	51 (27.1)	22 (11.7)	0.039	0.047
	Yes	115	63 (33.5)	52 (27.7)		
**Radiation**	No	169	105 (55.6)	64 (33.9)	0.139	0.153
	Yes	20	9 (4.8)	11 (5.8)		
**Concurrent Chemotherapy and Radiotherapy**	No	176	107 (56.6)	69 (36.5)	0.621	0.770
	Yes	13	7 (3.7)	6 (3.2)		
**Myometrium Invasion**	<1/2	66	43 (22.9)	23 (12.2)	0.299	0.350
	≥1/2	122	70 (37.2)	52 (27.7)		
**Property of Surgical Margin**	No	176	108 (57.1)	68 (36.0)	0.279	0.379
	Yes	13	11 (3.2)	2 (3.7)		
**Infiltration of Parauterine Organ**	No	179	108 (57.1)	71 (37.6)	0.983	1.000
	Yes	10	6 (3.2)	4 (2.1)		
**Lymphovascular Space Involvement**	No	173	107 (56.6)	66 (34.9)	0.157	0.186
	Yes	16	7 (3.7)	9 (4.8)		

**Table 3:** Correlation between *NR2F6* expression and the clinicopathological characteristics of early-stage cervical cancer.

Variable	NR2F6 Expression	
	Spearman’s Correlation Coefficient	*p*-Value
Age	0.015	0.837
Histological type	−0.047	0.523
HPV infection	0.083	0.256
FIGO Stage	0.198	0.006
Pelvic lymph node metastasis	0.397	<0.001
Squamous cell carcinoma antigen, ng/mL	0.211	0.006
Tumor size	0.003	0.996
Recurrence	0.232	0.001
Vital status	0.315	<0.001
Differentiation grade	−0.150	0.042
Chemotherapy	0.150	0.039
Radiation	0.108	0.140
Concurrent chemotherapy and radiotherapy	0.036	0.623
Myometrium invasion	0.076	0.301
Property of surgical margin	0.079	0.282
Infiltration of parauterine organ	0.002	0.983
Lymphovascular space involvement	0.103	0.158

**Table 4:** Cox regression univariate and multivariate analyses of prognostic factors in early-stage cervical cancer.

Variable	Univariate Analysis			Multivariate Analysis		
	No. Patients	*p*	Regression Coefficient (SE)	*p*	Relative Risk	95% Confidence Interval
**NR2F6**						
Low expression	114	<0.001	7.688 (0.447)	0.006	4.439	1.533–12.855
High expression	75					
**Pelvic lymph Node Metastasis**						
Absent	135	0.001	3.326 (0.374)	0.486	1.354	0.576–3.183
Present	54					
**Squamous Cell Carcinoma Antigen, ng/mL**						
≤1.5	110	0.006	3.065 (0.408)	0.449	0.689	0.262–1.808
>1.5	58					
**FIGO Stage**						
Ib1	72	0.010	1.551 (0.439)	0.082	1.484	0.951–2.315
Ib2	37					
IIa1	54					
IIa2	26					
**Recurrence**						
No	170	<0.001	30.659 (0.406)	0.000	21.311	7.741–58.667
Yes	19					
